# A Patient with Non-Hodgkin Lymphoma and Nonspecific Interstitial Pneumonia during Ibrutinib Therapy

**DOI:** 10.1155/2017/5640186

**Published:** 2017-11-10

**Authors:** Sven Jungmann, Wolf-Dieter Ludwig, Nicolas Schönfeld, Torsten-Gerriet Blum, Claudia Großwendt, Christian Boch, Beate Rehbock, Sergej Griff, Alexander Schmittel, Torsten T. Bauer

**Affiliations:** ^1^Department of Pneumology, Lungenklinik Heckeshorn, HELIOS Klinikum Emil von Behring, Berlin, Germany; ^2^Drug Commission of the German Medical Association, Herbert-Lewin-Platz 1, Berlin, Germany; ^3^Department of Radiology, HELIOS Klinikum Emil von Behring, Berlin, Germany; ^4^Diagnostic Radiology, Thoracic Imaging, Bismarckstr 45-47, Berlin, Germany; ^5^Institute of Pathology, HELIOS Klinikum Emil von Behring, Berlin, Germany; ^6^Onkologie Seestrasse, Seestr. 64, 13347 Berlin, Germany

## Abstract

We present a 74-year-old male with nonspecific interstitial pneumonia (NSIP) during treatment with ibrutinib for mantle cell lymphoma. Previously, the patient had received six cycles of bendamustine and rituximab and six cycles of R-CHOP, followed by rituximab maintenance therapy. Respiratory tract complications of ibrutinib other than infectious pneumonia have not been mentioned in larger trials, but individual case reports hinted to a possible association with the development of pneumonitis. In our patient, the onset of alveolitis that progressed towards NSIP together with the onset of ibrutinib treatment suggests causality. One week after ibrutinib was discontinued, nasal symptoms resolved first. A follow-up CT showed a reduction in the reticular hyperdensities and ground-glass opacities, suggestive of restitution of the lung disease. To our knowledge, this is the first case showing a strong link between ibrutinib and interstitial lung disease, strengthening a previous report on subacute pneumonitis. Our findings have clinical implications because pulmonary side effects were reversible at this early stage. We, therefore, suggest close monitoring for respiratory side effects in patients receiving ibrutinib.

## 1. Introduction

Ibrutinib, a selective Bruton's tyrosine kinase inhibitor, was approved initially in November 2013 by the US Food and Drug Administration for the treatment of mantle cell lymphoma and in February 2014 for B-cell chronic lymphocytic leukaemia, and later on also for Waldenström macroglobulinemia, all after first-line treatment [1]. Respiratory tract complications other than infectious pneumonia have not been mentioned in larger trials. However, a single case [2] and a case series [3] of four cases of ibrutinib-induced pneumonitis were published in May 2015 and February 2016, respectively. By November 2016, two cases of pneumonitis were mentioned on http://www.pneumotox.com [4].

## 2. Patient

We present a 74-year-old male whom we evaluated for a suspected nonspecific interstitial pneumonia (NSIP) under ibrutinib (560 mg QD). He was diagnosed with mantle cell lymphoma (2011) and received six cycles of bendamustine and rituximab (April to August 2011) and later six cycles of R-CHOP (October 2013 to February 2014), followed by rituximab maintenance therapy (February 2014 to January 2015). In February 2015, ibrutinib therapy was initiated because of abdominal progression. He responded well to ibrutinib with a partial remission. The patient felt healthy and denied cough, pain, dyspnoea, fever, night sweats, weight loss, nausea, or fatigue. However, he noted scales in his nasal discharge and frequent nasal bleeding. Medical history included hypertension, hyperlipidemia, peripheral artery occlusive disease, benign prostatic hyperplasia, and cigarette smoking (15 pack-years) until 35 years ago. He suffered from pneumonia at age 16 and received coronary artery bypass surgery at 65 years. Additional medication included once-daily doses of acetylsalicylic acid (100 mg), enalapril (10 mg), amlodipine (5 mg), hydrochlorothiazide (25 mg), and simvastatin (40 mg). There was no evidence of existing allergies or toxic exposures.

## 3. Physical Examination

The male patient was well nourished and was in no apparent distress, alert, and fully oriented. He had normal vital signs and no lymphadenopathy or thyromegaly. Lungs were clear to auscultation. He showed regular heart rate and rhythm without murmurs. He presented with soft abdomen, neither tender nor distended, normal bowel sounds, and no hepatosplenomegaly. No cyanosis, clubbing, rash, lesions, nor oedema were observed. Neurologic examination showed that cranial nerves II–XII were intact, and no focal sensorimotoric deficits were found. Skin examination showed no ulceration or induration, and joints and muscles were unaffected.

## 4. Diagnostic Findings

**Figure 1:**
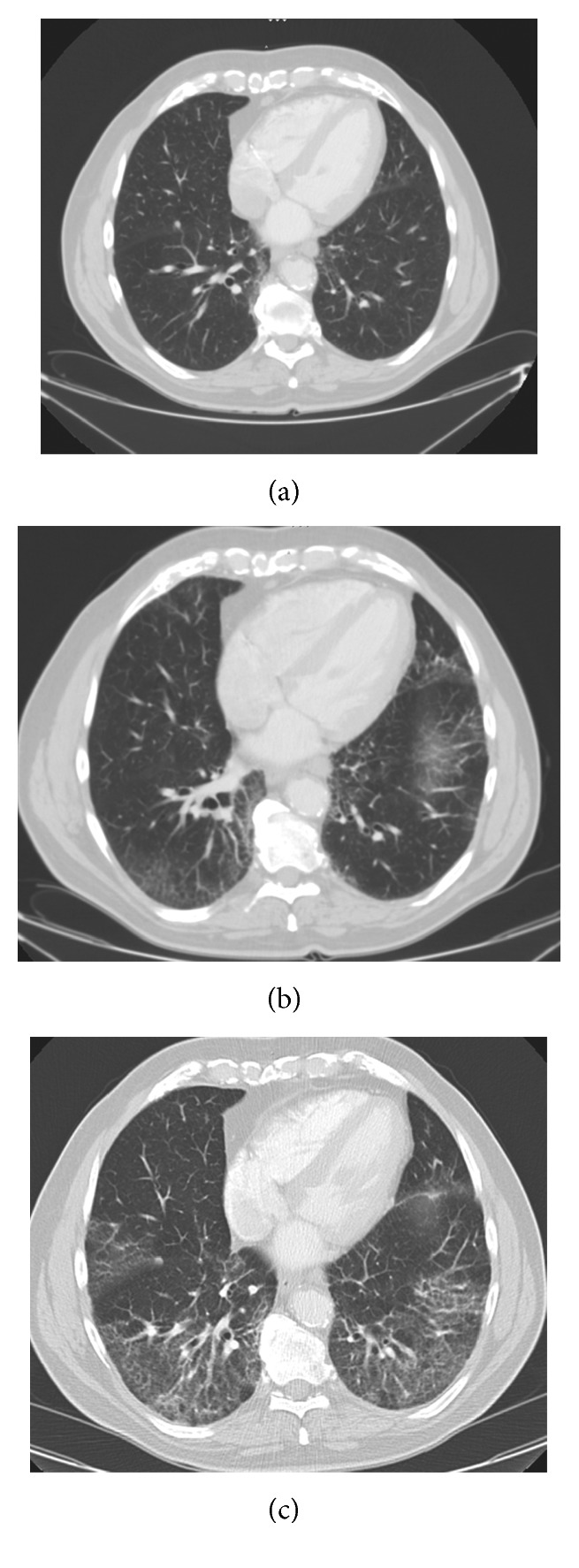
Computed tomography findings. (a) January 2015: the lower lung shows normal lung structures without signs of interstitial pneumonia or fibrosis. (b) June 2015: the same area now shows a bilateral mild interstitial pneumonia with ground-glass opacities. (c) March 2016: images show a more fibrotic structure with mild reticular abnormalities and ground-glass opacities.

An abdominal computed tomography (CT) revealed progressive non-Hodgkin lymphoma so that ibrutinib was initiated (October 2015). All abdominal CT scans included basal lung sections, which were normal until then. Subsequent CT scans depicted progressive ground-glass opacities in the middle and both lower lobes. In April 2015, a discrete fibrotic transition became visible, with radiologic patterns compatible with early fibrotic NSIP (Figure 1). Bronchoscopy revealed mild hypervascularization particularly of the upper lobe and main bronchi. Bronchoalveolar lavage (BAL) showed a lymphocytic alveolitis (47% lymphocytes) with a balanced CD4/CD8 ratio. Sampling of the subcarinal lymph node excluded local progression of the disease. The BAL was sent for a routine bacterial workup including Gram stains, and cultures with no potentially pathogenic microorganisms recovered.  Table 1 gives an overview of relevant clinical data and findings.

## 5. Literature Review

Our literature search (PubMed Central) identified articles cited up to November 21, 2016, using the keywords “ibrutinib,” “PCI 32765,” “imbruvica,” or “Bruton's Tyrosine Kinase Inhibitor” in combination with the keywords (Boolean “AND”) “pneumonia,” “interstitial lung disease,” “fibrotic,” or “pneumonitis” in title, abstract, and body, yielding 14 publications. Articles that did not address the issue of respiratory side effects in patients receiving ibrutinib were excluded, leaving seven relevant articles that describe pneumonia [1, 5–11] and one case series that describes pneumonitis as a side effect of ibrutinib [3], which also quotes another case presented in a conference poster publication [2]. However, NSIP has not been described to date. Two cases of pneumonitis but no association of NSIP with ibrutinib were described on http://www.pneumotox.com [4]; however, pneumonitis is mentioned twice. The Drug Commission of the German Medical Association has received 236 reports of adverse effect suspicions for ibrutinib until May 2016, two of which include atypical pneumonia and a total of 21 reported pneumonia [12].

## 6. Discussion

Only a few ibrutinib-associated lower respiratory tract side effects are known. In seminal phase 2 [13] and phase 3 trials [14], ibrutinib was shown to be effective and well tolerated with no dose-limiting toxicity being observed. Postmarketing adverse effect studies revealed the following pulmonary side effects: a study [6] involving 51 patients with chronic lymphocytic leukaemia receiving ibrutinib described 12% grade 2 and 6% grade 3 pneumonia and pulmonary infiltrates in 2% as treatment-related adverse events and concluded that ibrutinib's safety profile was encouraging. A long-term follow-up [9] of 111 patients reported a favourable benefit-risk profile. While dyspnoea was seen in 32%, two-thirds involved respiratory function, such as chronic obstructive pulmonary disease, respiratory infections, malignant pleural effusion, and pneumonia (8%, grade ≥ 3). Another study [10] including 132 patients concluded that pneumonia was among the most common complications and resumed that ibrutinib is well tolerated. In all studies, complications were well manageable with little or no chronicity.

Our patient's association of the alveolitis that progressed into NSIP pattern after the commencement of ibrutinib treatment suggests causality. One week after the ibrutinib was discontinued, nasal symptoms resolved. In June 2016, a follow-up CT showed a diminution of the reticular hyperdensities and ground-glass opacities with minimal residues in the lingual and lower lobe. To our knowledge, this is the first case showing a link between ibrutinib and fibrotic lung disease, strengthening the previous report on subacute pneumonitis [3]. Similar to our patient, radiographic changes in the four cases presented by Mato et al. [3] were detected after commencement of ibrutinib therapy and resolved after stopping ibrutinib, and respiratory symptoms recurred in the one case that was rechallenged with the drug. Our findings have clinical implications because pulmonary side effects were reversible at this early stage.

Since July 2017, the patient receives lenalidomide after an abdominal progress. Thus far, the patient does tolerate the medication well and is scheduled for restaging. We abstained to reintroduce ibrutinib into the therapeutic regimen due to the lack of information on the repeated reversibility of the pulmonary damage.

Some limitations in the clinical workup apply. We did not look for *Pneumocystis jirovecii* infection and did not exclude viral infection (e.g., CMV) as a cause of pulmonary infiltrates in this patient. However, a normal lactate dehydrogenase and the good clinical improvement with corticosteroids suggest neither one of these infections to be the cause of the clinical deterioration.

We, therefore, suggest monitoring patients receiving ibrutinib for respiratory side effects. Also, more research is required to define the underlying pathways that lead to pulmonary toxicities of signal transduction inhibitors: as Mato et al. point out [3], other targeted therapies such as rapamycin inhibitors [15, 16], phosphatidylinositol 3-kinase inhibitors [17], and spleen tyrosine kinase inhibitors [18] appear to cause similar pulmonary toxicities. Mato et al. suggest that the inhibition of signal transduction enhances the expression of proinflammatory cytokines and the innate immune system [3]. They further propose that Bruton's tyrosine kinase might serve as a mediator of lipopolysaccharide-induced dendritic cell maturation and macrophage polarisation and highlight that several studies have reported an increase in alveolar infiltration of T helper 2 proinflammatory cytokines in Bruton's tyrosine kinase-deficient mice, which results in airway inflammation [19, 20].

## Figures and Tables

**Table 1:** Pulmonary function test and relevant blood values (reference values in parentheses).

Test	Value
One-second forced expiratory volume	2.53 liters (81% of predicted)
Vital capacity	3.17 liters (75% of predicted)
Total lung capacity (single breath)	4.5 liters (62% of predicted)
FEV1/FVC ratio	79.84% (106% of predicted)
Single-breath Krogh transfer factor for carbon monoxide	1.05 mmol/(min kPa l) (84% of predicted)
NT-proBNP	293 ng/l (<229)
Estimated GFR	83 ml/min (>90)
Erythrocytes	4.3 G/l (4.5–5.9)
Haemoglobin	12.9 g/dl (14.0–17.5)
MCHC	32 g/dl (33–36)
Thrombocytes	108 G/l (139–335)
Neutrophils	1.47 G/l, 32% (1.6–7.1, 40–75%)
Monocytes	1.11 G/l, 24% (0.2–0.6, 4–11%)
IgG	5.73 g/l (7–16)
IgG4	0.002 g/l (0.052–1.250)
IgM	0.31 g/l (0.4–2.3)
Capillary pH	7.46 (7.37–7.45)
Capillary pCO_2_	34 mmHg (35–46)
INF-gamma	0.49 IU/ml (<0.35)
Relevant normal lab values included	ANA, c-ANCA, p-ANCA, anti-CCP, rheumatoid factor, IgA, IgG1-3, IgE, creatinine, and lactate dehydrogenase
